# Recent Advances in Biomembrane Models for Studying Interactions with Bio-/Molecules

**DOI:** 10.3390/membranes16050174

**Published:** 2026-05-12

**Authors:** Jadwiga Maniewska, Katarzyna Gębczak

**Affiliations:** 1 Department of Medicinal Chemistry, Faculty of Pharmacy, Wroclaw Medical University, Borowska 211, 50-556 Wroclaw, Poland; 2 Division of Basic Medical Sciences, Faculty of Pharmacy, Wroclaw Medical University, Borowska 211, 50-556 Wroclaw, Poland

## 1. Introduction

Biological membranes play a pivotal role in determining cellular organization and functionality, as they provide complex and dynamic environments for interactions with a diverse array of biomolecules and external compounds. The physicochemical properties of these membranes, shaped by factors such as lipid composition, membrane-associated proteins, and lateral heterogeneity, are critical for governing essential biological processes. Therefore, achieving a comprehensive understanding of these interactions is vital for advancing fields such as biophysics, pharmacology, and medicinal chemistry.

In recent years, significant progress has been made in developing biomembrane models and the methodologies employed to study them. Techniques such as calorimetry, fluorescence-based measurements, and high-resolution imaging, alongside molecular dynamics simulations, have opened avenues for increasingly precise characterization of membrane structures, dynamics, and mechanisms of interaction. At the same time, there has been a growing focus on how lipid diversity, domain formation, and cholesterol content influence membrane behavior and responsiveness to interacting molecules.

Nevertheless, several challenges persist. A key limitation is the difficulty in translating findings from simplified model systems to the inherently more complex environment of native biological membranes, which are characterized by compositional asymmetry, spatial heterogeneity, and continuous remodeling. Furthermore, the molecular mechanisms by which small molecules, peptides, and proteins disrupt membrane organization and initiate downstream biological effects remain inadequately understood, particularly regarding interconnected phenomena such as cytoskeletal rearrangement and lipid domain dynamics. Another unresolved issue is establishing predictive relationships between membrane-level interactions and pharmacological outcomes.

The Special Issue entitled “Recent Advances in Biomembrane Models for Studying Interactions with Bio-/Molecules,” published in *Membranes* (MDPI), addresses these challenges by presenting eight contributions that integrate experimental and computational approaches to explore membrane–molecule interactions across various organizational levels. These studies enhance our understanding of how membrane composition, molecular structure, and biophysical properties influence biological activity.

## 2. Overview of Special Issue Contributions

Several papers in this collection focus on the interaction of small bioactive molecules with model lipid membranes, combining physicochemical characterization with biological evaluation. In Contribution 1, arylpiperazine oxicam derivatives were investigated using differential scanning calorimetry (DSC) in phospholipid bilayers composed of 1,2-dimyristoyl-sn-glycero-3-phosphocholine (DMPC). The results showed concentration-dependent decreases in the phase transition temperature, indicating incorporation of the compounds into the lipid bilayer and partial disruption of lipid organization. Biological evaluation using MTT assays in cancer (MCF-7, MCF-7/DX, LOVO, and LOVO/DX) and normal (V79) cell lines revealed moderate cytotoxic activity, particularly against colorectal cancer cells, whereas cyclooxygenase inhibition remained weak despite molecular docking, suggesting potential interactions with the COX-2 active site. These findings indicate that membrane interactions constitute a primary determinant of the observed biological activity.

In a related study (Contribution 4), another series of arylpiperazine oxicam derivatives was evaluated using a combined experimental and theoretical approach. Cyclooxygenase inhibition assays, cytokine mRNA expression analysis, and antioxidant activity tests based on DPPH and ABTS radical scavenging were complemented by density functional theory calculations to assess reactivity profiles. The results demonstrated moderate to high selectivity toward the COX-2 isoform, low cytotoxicity relative to reference compounds, and significant antioxidant capacity. The observed interactions with phospholipid membranes further emphasize the importance of membrane-related effects in determining pharmacological behavior.

Natural compounds and plant-derived molecules are addressed in detail in several contributions. In Contribution 2, polyphenolic fractions enriched in flavonoid glycosides and caffeoylquinic acids, derived from *Inula oculus-christi* L. (medicinal plant), were investigated using confocal microscopy. Membrane order was assessed with the fluorescent probe Di-4-ANEPPDHQ, while actin cytoskeleton organization was visualized using TRITC-phalloidin staining. The results revealed distinct cell-specific effects, with flavonoid glycosides inducing membrane ordering in cancerous A549 cells and membrane fluidization in non-malignant MDCK II cells, accompanied by pronounced reorganization of F-actin structures. These findings demonstrate a strong coupling between membrane biophysical properties and cytoskeletal dynamics, as well as the influence of molecular structure on membrane interactions.

Complementary insight at the molecular level is provided in Contribution 3, in which the author employed molecular dynamics simulations to investigate the behavior of nymphaeol A (isolated from propolis produced by honeybees) in complex lipid membranes. The simulations demonstrated spontaneous insertion of the molecule into the bilayer, preferential localization near the carbonyl region of phospholipids, and a tendency to adopt an extended conformation with an average tilt angle of approximately 35° relative to the membrane normal. The compound exhibited significant mobility along the membrane normal and showed preferential interactions with specific lipid species, including POPC and PSM, while slightly increasing membrane fluidity. These results provide detailed insight into the relationship between molecular structure, membrane localization, and functional activity.

The importance of lipid composition as a key determinant of membrane interactions is further emphasized in Contribution 5, in which the authors examined the interaction of high-density lipoprotein (HDL) particles with model membranes using fluorescence-based techniques. In the study, they analyzed lipid domain organization, membrane order, and both lateral and intramolecular mobility, with a particular focus on the glycerol region of lipids and the associated hydrogen bond network. The results demonstrated that both fluid-phase and certain gel-phase membranes interact with HDL particles, although with distinct functional outcomes: gel-phase membranes favor binding, whereas fluid-phase membranes facilitate cargo transfer. Increased glycerol region mobility and a strengthened hydrogen bond network were associated with enhanced anchoring interactions, while reduced water structuring promoted lipid transfer. The presence of cholesterol was shown to reduce HDL interaction by increasing membrane packing density and stiffness, thereby modulating membrane fluidity.

Two review articles provide broader perspectives on membrane-mediated biological processes and their pharmacological implications. Contribution 6 addressed the role of biological membranes in amyloidogenic protein toxicity, emphasizing how membrane physicochemical properties influence protein misfolding, aggregation, and cytotoxicity. In the review, the authors discuss small molecules capable of intercalating into lipid bilayers, including aminosterols, cholesterol, ubiquinone, and selected polyphenols, which can modulate membrane properties and reduce amyloid–membrane interactions. At the same time, inconsistencies in reported protective effects highlight the need for a deeper mechanistic understanding of these processes.

Contribution 7 focused on the role of membrane transporters, particularly ATP-binding cassette (ABC) and solute carrier (SLC) proteins, in drug pharmacokinetics. These transporters play a crucial role in drug absorption, distribution, and elimination. The complex interplay between transporter expression, physiological conditions, and co-administered compounds is identified as a key factor influencing drug efficacy and safety.

Finally, the authors of Contribution 8 examined the influence of cholesterol on membrane-targeted bioactive peptides. They highlight how cholesterol modulates lipid packing, membrane rigidity, and lateral mobility, thereby affecting peptide insertion, membrane disruption, and selectivity. The findings emphasize the importance of incorporating realistic membrane compositions, including cholesterol and sphingolipids, into experimental models in order to better replicate native biological systems.

## 3. Conclusions

The studies compiled in this Special Issue highlight the pivotal role of biomembrane models in elucidating the mechanisms governing membrane–molecule interactions. Taken together, the contributions demonstrate that membrane composition, structural organization, and dynamic properties critically influence molecular behavior and, consequently, biological function in systems ranging from small bioactive compounds to complex biological assemblies.

Importantly, the presented works contribute to narrowing several gaps that persist in the field. They provide mechanistic evidence that membrane interactions can act as primary drivers of biological activity, clarify the relationship between membrane organization and cellular processes, and emphasize the regulatory role of lipid composition, including cholesterol and specific lipid classes, in modulating these effects. Furthermore, the combined use of experimental techniques and computational modeling underscores the importance of multiscale approaches for achieving a more comprehensive description of membrane-associated phenomena.

However, several challenges remain. Future research should focus on developing membrane models that more accurately represent the structural and compositional complexities of native systems, including lipid asymmetry, heterogeneity, and curvature effects. Stronger integration of experimental findings with theoretical frameworks will also be essential for establishing predictive models that link molecular interactions to functional outcomes.

From a practical perspective, translating these fundamental insights into real-world applications is a key objective. Future studies should prioritize the rational design of membrane-active compounds, optimization of drug delivery strategies, and identification of therapeutic targets related to membrane interactions. Enhancing our understanding of membrane–molecule interactions in complex biological contexts will be vital for tackling issues such as multidrug resistance and targeted therapies.

This Special Issue aims to serve as a valuable reference and inspire further research that connects model membrane systems with the intricacies of biological membranes, ultimately advancing the field of biomembrane science.

